# Electrochemical impacts of sheet-like hafnium phosphide and hafnium disulfide catalysts bonded with reduced graphene oxide sheets for bifunctional oxygen reactions in alkaline electrolytes†

**DOI:** 10.1039/c8ra09598a

**Published:** 2019-01-18

**Authors:** Mayilvel Dinesh Meganathan, Taizhong Huang, Hengyi Fang, Jianfeng Mao, Guoxin Sun

**Affiliations:** School of Chemistry and Chemical Engineering, University of Jinan 336 West Road of Nanxinzhuang Jinan Shandong 250022 China chm_huangtz@ujn.edu.cn; Institute for Superconducting & Electronic Materials, Australian Institute for Innovative Materials, University of Wollongong Wollongong NSW 2522 Australia jmao@uow.edu.au

## Abstract

Non-noble metal-based catalysts with efficient catalytic activities for the oxygen evolution reaction (OER) and oxygen reduction reaction (ORR) are critical for energy conversion devices, including fuel cells and metal–air batteries. In this work, novel hafnium phosphide-reduced graphene oxide nanosheets (HfP-rGO NS) and hafnium disulfide-reduced graphene oxide nanosheets (HfS_2_-rGO NS) were synthesized and investigated as bifunctional electrocatalysts for OER and ORR. The prepared HfP-rGO NS and HfS_2_-rGO NS catalysts showed nanosheet structures, where the HfP or HfS_2_ nanosheet was closely packed with rGO. A unique methodology was adopted to lodge the non-metal oxide catalytic sheets (*i.e.*, HfP and HfS_2_) over the rGO sheets, which positioned the oxide layer on the catalytic sheet surface for instant oxygen evolution. Low intensity X-ray diffraction patterns and Raman spectra confirmed the sheet-like structure of HfP-rGO NS and HfS_2_-rGO NS. Scanning electron microscope mapping images revealed that all elements (*i.e.*, Hf, P, C and O for HfP-rGO NS and Hf, S, C and O for HfS_2_-rGO NS) were equally distributed in the synthesized heteroatomic nanosheets. Moreover, both the HfP-rGO NS and HfS_2_-rGO NS demonstrated excellent durability for both ORR and OER. This outperforms the most state-of-the-art non-precious-metal-based bifunctional catalysts, which is attributed to the synergistic effect of rGO and Hf-based catalysts. The different ORR and OER reaction potentials in HfP-rGO NS and HfS_2_-rGO NS likely result from the influence of HfP and HfS_2_.

## Abstract 2

Sheet-like structured hafnium phosphide (HfP) and hafnium disulphide (HfS_2_) catalysts show high catalytic activity for both oxygen evolution and oxygen reduction reactions, which are determined by the compositional anion ions.

## Graphical abstract

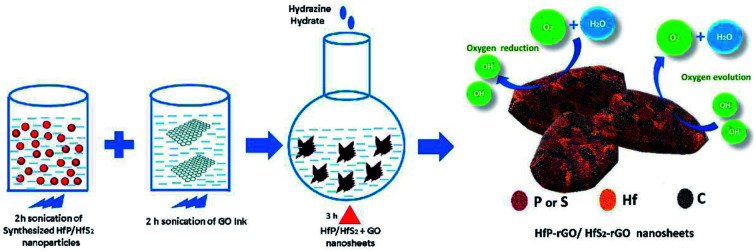

## Introduction

Renewable electrochemical energy conversion technologies, such as fuel cells and metal–air batteries, are in high demand in today's society in order to reduce environmental pollution and to act as a replacement for fossil fuels.^1–5^ In general, fuel cells are primary energy devices that generate electricity *via* a redox reaction between the fuel anode and oxygen cathode. Reversible fuel cells are similar secondary energy devices that can reduce oxygen (O_2_) and, in reverse, produce O_2_. Therefore, the oxygen evolution reaction (OER; 4OH^−^ → O_2_ + 2H_2_O + 4e^−^) and the oxygen reduction reaction (ORR; O_2_ + 2H_2_O + 4e^−^ → 4OH^−^) are the two vital electrochemical reactions at the oxygen cathode that drive these devices.^6–8^ However, the slow and sluggish kinetics of these oxygen reactions at the air cathode result in high overpotential and poor oxygen adsorption, which severely limit the overall efficiency of the working cells.^9–16^ Therefore, designing bifunctional electrocatalysts for both OER and ORR is highly desirable for reversible fuel cell and metal–oxygen battery systems.

Until now, many research articles have showcased electrocatalysts for either ORR or OER; but only a few reports on both ORR and OER have been published. Noble metals, such as Pt, Ru, Rh, Pd and Au, for example, have been reported as promising and highly efficient electrocatalysts. Among them, Pt is the most well-known active electrocatalyst for the ORR, but is limited in terms of the OER because of the formation of Pt oxide on the catalyst surface.^14,17^ Due to the high cost of noble metals, transition-metal-based compounds such as metal oxides, metal carbides, metal nitrides, metal sulfides and metal phosphides have been utilized as alternative catalysts because they are abundant, cheap, electrochemically active and chemically stable.^17–20^ Among them, metal oxides such as Co_3_O_4_, NiO and MnO_2_ have been considered as the most promising alternatives due to the easy transfer of O^2−^ from the metal–oxygen bonds. Nevertheless, reported metal oxide catalysts still suffer from high overpotential, low catalytic activity and poor cycling stability.^9,14^ Meanwhile, most of the metal oxides and hydroxides have been used only for the OER.^21^ Compared with transition metal oxides, transition metal sulfides usually possess better electronic conductivity. Considering their cost, performance stability and safety issues, the metal sulfides are very attractive for electrochemical applications.^22–24^ Similarly, the metal phosphides are also capable of excellent catalytic performance and durability, and also have higher electrical conductivity than oxides. In particular, metal phosphides are used for OERs by the virtue of their proton-acceptor characteristics which allows them to form molecules easily (*i.e.*, oxygen, hydrogen *etc.*).^25,26^

In addition, metal-based electrocatalysts and carbon-based materials have also been employed as bifunctional active catalysts due to their good intrinsic OER reactivity and corrosion resistivity at high oxidation potentials. Meanwhile, doping heteroatoms to nanocarbon materials can lead to the favorable formation of OH^−^*via* the direct 4e^−^ pathway, and could significantly boost ORR activity. Oxygen reduction catalysis *via* doped N, S, P, B or I on dual-atom-doped carbon nanotubes (CNTs) and/or graphene materials has been studied broadly.^14^ This is because the heteroatoms can tune the electronic properties of C and offer more active sites.^6,27^ In particular, reduced graphene oxide (rGO) attracting the interest due to its few-layer or multi-layer atomic carbon sheets, as well as the presence of both C and O (heteroatoms), which make them more favorable for electrochemical reactions. Moreover, the catalytic performance of graphene-based materials could be further improved by incorporating transitional metals. For example, electrocatalysts such as Co_3_O_4_/N-rmGO and Co_4_N/rGO have been discussed as highly active catalysts for the ORR because of their well-assorted heteroatoms.^8,28^ The combination of rGO sheets with metallic compounds (*i.e.*, heteroatom doping) can improve the electronic conductivity and increase the surface area, and hence make the electrocatalyst highly active due to the synergistic effects.^14,29,30^ Similarly, many active heterogeneous catalysts, such as FeP@NPC,^31^ FeP@PNC-800,^32^ Fe_*x*_P/NPCS,^25^ rGO–Co–Pi,^33^ N-CG-CoO,^1^ V(C,N),^34^ N-Co_9_S_8_/G^35^ and FeS NS,^36^ have been reported for the ORR/OER. However, the need remains to achieve high-performance bifunctional electrocatalysts in bulk applications for energy conversion devices based on oxygen electrocatalysis.

In this work, hafnium disulfide-reduced graphene oxide nanosheets (HfS_2_-rGO NS) and hafnium phosphide-reduced graphene oxide nanosheets (HfP-rGO NS) were electrochemically tested for the OER and ORR in 0.1 M KOH electrolyte solution, and their intrinsic bifunctional behavior for oxygen reactions was demonstrated. In addition, instead of anchoring the catalytic nanoparticles on the rGO sheets, this work combines the catalytic (Hf) nanosheets with rGO sheets for better stability and high synergistic effect during the oxygen reactions. Interestingly, both HfP-rGO NS and HfS_2_-rGO NS are competitive with each other in different aspects of the OER and ORR electrochemical measurements.

## Experimental methods

### Preparation of HfP-rGO and HfS_2_-rGO nanosheets

HfP-rGO NS were prepared by the following procedure. 150 mg of red phosphorous was dissolved in 50 ml of ethylene glycol under constant stirring. After fine dissolution, 150 mg of hafnium tetrachloride (HfCl_4_) was added pinch-by-pinch into the stirring solution. The solution mixture was ultra-sonicated for 1 h and moved to a Teflon flask to be heated at 200 °C in the microwave oven for 15 h. After heating, the solution mixture was cooled naturally, and then the dark red-colored HfP slurry was collected and washed with ethanol.^37^ The obtained HfP slurry was dissolved in 50 ml of ultra-pure (UP) water and sonicated for 2 h. In parallel, 17 ml of GO ink (6 mg ml^−1^) was dissolved in 30 ml of UP water and sonicated for 2 h. The GO ink was prepared by the modified-Hummers' method.^8^ After 2 h, the sonicated GO solution was added dropwise into the sonicating HfP solution. After that, the physically mixed HfP-GO nanosheet solution was sonicated for a further 30 min and then transferred into a round-bottom flask. Next, 3 ml of hydrazine hydrate (H_4_N_2_·H_2_O) was dropped into the HfP-GO solution, and then the round-bottom flask was refluxed for 3 h at 120 °C in an oil bath. After the chemical reduction, dark red-colored HfP-rGO NS slurry was washed with ethanol and UP water separately. Then the slurry was completely dried at 80 °C and the HfP-rGO NS catalyst powder was collected and finely ground.

The above procedure was also followed to prepare HfS_2_-rGO NS, except that 300 mg of Na_2_S crystals and 150 mg of HfCl_4_ were used in the 24 h solvothermal treatment at 200 °C.^38^ In this process, black-colored HfS_2_-rGO NS powder was obtained.

### Material characterization

Crystals of the prepared HfS_2_-rGO NS and HfP-rGO NS catalysts were analyzed by powder X-ray diffraction (XRD) using a Bruker D8 advanced diffractometer with Cu Kα radiation (*λ* = 1.5418 Å). Raman spectra of HfS_2_-rGO NS and HfP-rGO NS were recorded using a Renishaw inVia spectrometer with 532 nm laser excitation. Morphologies of the HfS_2_-rGO NS and HfP-rGO NS catalysts were captured by scanning electron microscopy (SEM), and the elements in the catalysts were distinguished by elemental mapping techniques using a Hitachi (S-4800) SEM. The in-depth morphology of the prepared catalysts was further examined by transmission electron microscopy (TEM) and selected area electron diffraction tests using a Tecnai (20 U-TWIN) TEM. The elemental composition and the binding energies of the HfS_2_-rGO NS and HfP-rGO NS were detected using a Thermo Fisher Scientific ESCALAB 250Xi X-ray photoelectron spectrometer (XPS) with Al Kα radiation.

### Electrochemical measurements

All the ORR and OER electrochemical measurements of the prepared HfS_2_-rGO NS and HfP-rGO NS catalysts were carried out using a CHI 760D electrochemical workstation. For ORR measurements, cyclic voltammetry (CV), linear sweeping voltammetry (LSV), the construction of Tafel plots, electrochemical impedance spectroscopy and current–time (*i*–*t*) chronoamperometric tests were conducted using a three-electrode half-cell set-up (glassy carbon disk-working electrode, graphite rod-counter electrode and Ag/AgCl-reference electrode) in 0.1 M KOH electrolyte solution. For N_2_ and O_2_-saturated electrolytes, N_2_ and O_2_ gases were bubbled into the 0.1 M KOH electrolyte for 1 h each. CV curves were recorded in N_2_-saturated electrolyte at a sweeping rate of 20 mV s^−1^. A forward scanning potential range from 0.2 V to −0.8 V (1.16 V to 0.16 V in reversible hydrogen electrode [RHE]) was maintained for the ORR. Afterwards, the O_2_ was passed into the electrolyte, and then CV, LSV, Tafel, AC impedance and amperometric *i*–*t* curve tests were conducted. Similarly, a rotating ring-disk electrode (RRDE) and 3A electrode were used for the rotating disk electrode (RDE) and RRDE tests, respectively, in N_2_- and O_2_-saturated 0.1 M KOH electrolyte. In the RDE tests, CV curves were first recorded in N_2_-saturated 0.1 M KOH, then the CV and LSV were conducted in O_2_-saturated electrolyte. The LSV curves were recorded at a sweeping rate of 5 mV s^−1^ with a disk rotating speed in the range of 400, 625, 900, 1225, 1600 and 2025 rpm. In the RRDE tests, the LSV test at 5 mV s^−1^ was recorded at a rotating speed of 1600 rpm using the RRDE.

For OER measurements, the O_2_ gas was bubbled in 0.1 M KOH for 1 h and all the electrochemical tests were carried out with the RDE electrode with 1600 rpm continuous rotation. At first, the LSV test was conducted at 5 mV s^−1^ in the reverse scan potential ranges from 0 V to 1.2 V (0.96 V to 2.16 V in RHE), then Tafel, AC impedance and amperometric *i*–*t* curve tests were conducted for OER. In order to prepare the catalyst layer on the electrode, 3 mg of either HfS_2_-rGO NS or HfP-rGO NS was mixed with 30 μl of Nafion and 270 μl of UP water (18.25 MΩ) and sonicated for 1 h to obtain the catalyst ink. Then, 5 μl (10 μl for the RRDE electrode) of the catalyst ink was pipetted onto the working electrode. All working electrodes were polished and washed with ethanol and deionized water before the catalyst was loaded.

## Results and discussion

### Structural characterization

**Fig. 1:**
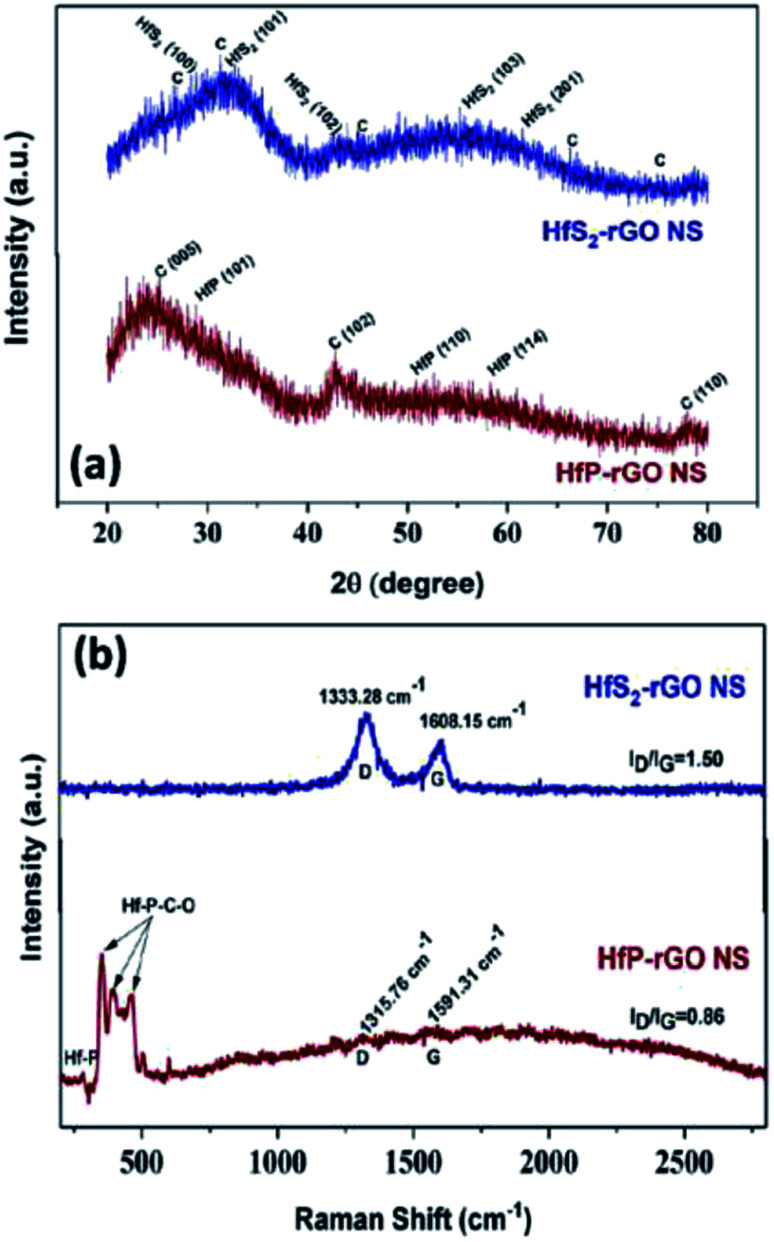
(a) X-ray diffraction patterns and (b) Raman spectra of HfS_2_-rGO nanosheets and HfP-rGO nanosheets.

XRD patterns for the prepared HfS_2_-rGO NS and HfP-rGO NS catalysts are shown in Fig. 1a, revealing that sharp intense peaks were not found due to the non-crystalline, sheet-like structures. For HfS_2_-rGO NS, a broad peak ranging from 20° to 40° and comprising HfS_2_ and C is seen, in which the high intensity is due to the presence of C. The presence of HfS_2_ is confirmed by tiny peaks at 28.2° (100), 32.3° (101), 42.2° (102), 55.4° (103) and 61.3° (201), which are indexed to JCPDS file no. 28-0444. Tiny diffraction peaks of C were detected at 31.2°, 26.6°, 45.6°, 66.1° and 75.3° (JCDPS no. 46-0943). The HfP-rGO NS have the highest intense peak due to C (005) at 25.3°. After C (005), the peak starts to fall gradually until 40°, after which HfP (101) is seen at 29.1°. C (102) at 42.8° results in a sharp peak, which confirms the confinement of the carbon atomic sheet from GO. Furthermore, HfP (110) and HfP (114) are identified by small peaks at 50.7° and 58.2°, respectively (JCPDS no. 65-3506). At 78°, a sharp peak for C (110) is seen that is indexed to JCPDS no. 26-1077. The XRD patterns of the HfS_2_-rGO nanosheets shows a broad hump, which can be mainly attributed to sulfide (HfS_2_) and rGO. Also, the weak C peaks indicate the nanosheets with thick rGO layers. For HfP-rGO nanosheets, the oxide-influenced humps are not found; instead, sharp C peaks are seen due to the thin carbon sheets. Fig. 1b shows the Raman spectra of HfP-rGO NS and HfS_2_-rGO NS. The D and G peaks of the HfS_2_-rGO nanosheets are positioned at 1333.28 cm^−1^ and 1608.15 cm^−1^, respectively. The intensity ratio of the D and G bands (*i.e.*, *I*_D_/*I*_G_ for HfS_2_-rGO NS) is 1.50. *I*_D_/*I*_G_ ≫ 1 indicates that the intensity of the D band was higher than that of the G band, which means that more structural defects exist in the rGO.^8,39^ The Raman spectra of HfP-rGO NS show a peak at 285.15 cm^−1^, which can be attributed to Hf–P. Strong intense peaks at 358.41 cm^−1^, 392.77 cm^−1^ and 467.55 cm^−1^ are attributed to the effect of heteroatomic elements such as C and O, which influence the phosphide (Hf–P–C–O), while forming the homogeneous HfP-rGO nanosheets.^40^ The D and G peaks for HfP-rGO NS are obtained at 1315.76 cm^−1^ and 1591.31 cm^−1^, respectively. Here, *I*_D_/*I*_G_ = 0.86 (*i.e.*, minimal structural defects) occurred on the C atoms. The Raman spectra reveal that HfP-rGO NS is atomically thin and consists of low structure defects in the carbons.^41^

**Fig. 2:**
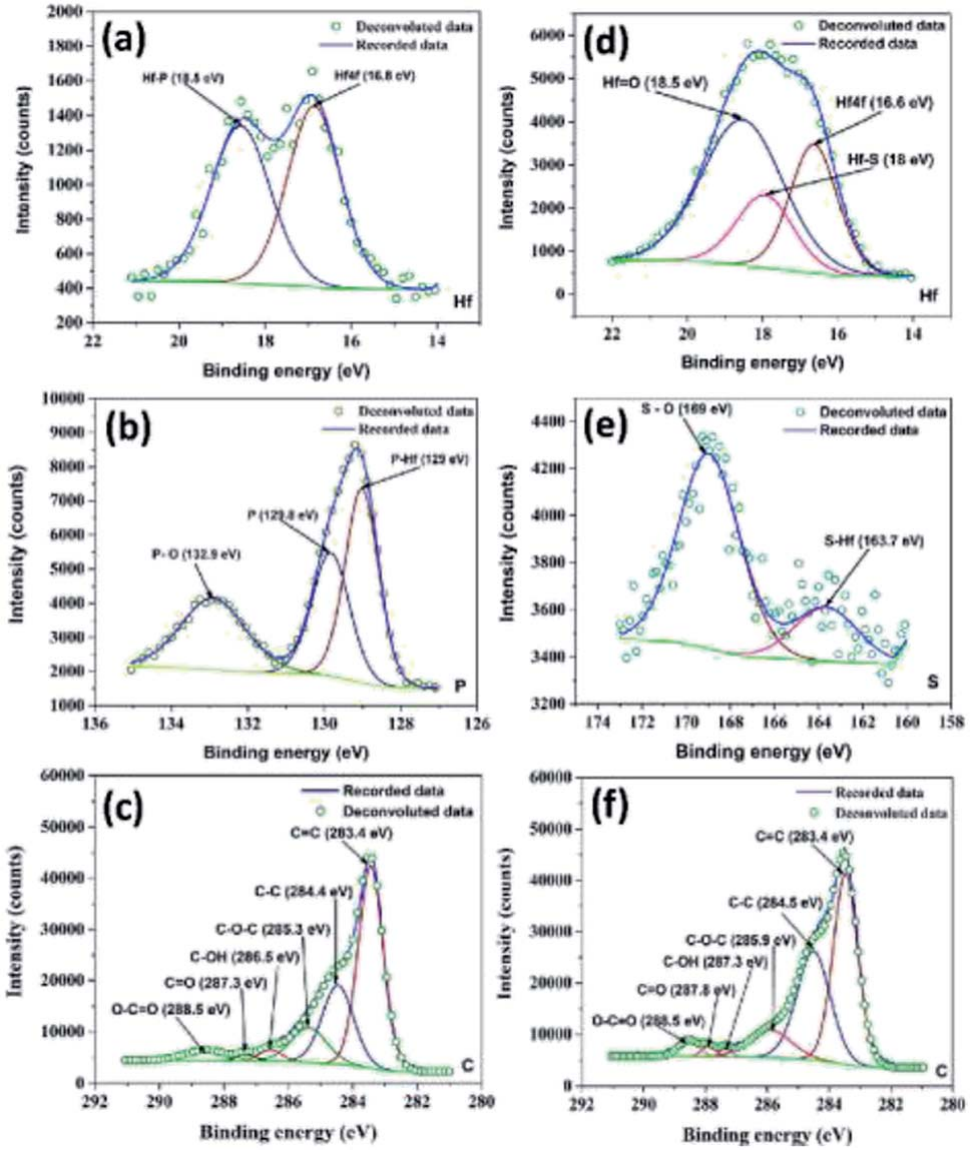
High-resolution XPS spectra of (a) hafnium, (b) phosphorus and (c) carbon for HfP-rGO nanosheets. High-resolution XPS spectra of (d) hafnium, (e) sulfur and (f) carbon for HfS_2_-rGO nanosheets.

Meanwhile, XPS spectra of the as-prepared HfP-rGO NS and HfS_2_-rGO NS catalysts are shown in Fig. S1a and c in the ESI.† The high-resolution XPS of Hf for HfP-rGO NS is shown in Fig. 2a. The Hf 4f peak at 16.8 eV and Hf–P at 18.5 eV form twin peaks for Hf in HfP-rGO NS. The equivalent phosphide peak (Hf–P) rising next to Hf 4f shows the presence of Hf and phosphide formation in the prepared HfP-rGO NS. Similarly, high-resolution Hf XPS of HfS_2_-rGO NS is shown in Fig. 2d, in which Hf 4f is visible at 16.6 eV along with a sulfide peak (Hf–S) at 18 eV and an oxide peak (Hf=O) at 18.5 eV, combining to form a single peak. Here, the intensity of the Hf=O peak is higher than that of Hf 4f and Hf–S, which should be increased by the reaction of rGO. However, in the HfP-rGO NS, the oxide effects are substantially controlled by elemental phosphorus. Fig. 2b shows the high-resolution XPS of phosphorus for HfP-rGO NS, in which the elevated peak at 129 eV corresponds to P–Hf, and an associated peak at 129.8 eV is attributed to elemental phosphorus. A small peak at 132.9 eV may be attributed to P–O. In parallel, the high-resolution XPS for sulfur in HfS_2_-rGO is shown in Fig. 2e. A small peak at 163.7 eV shows the presence of HfS_2_, whereas the S–O peak is found at 169 eV. The comparison of the sulfur XPS for HfS_2_-rGO NS and phosphorus XPS spectra for HfP-rGO NS clearly demonstrates that the oxide effect from rGO has a minimal influence on the HfP, whereas HfS_2_ failed to control. The C XPS for HfP-rGO NS and HfS_2_-rGO NS are shown in Fig. 2c and f, respectively. The C=C peak at 283.4 eV in both the HfP-rGO and HfS_2_-rGO NS catalysts are attributed to the sp^2^ carbon bond (C=C) of rGO. Next, the C–C peak is visible at 284.4 eV for HfP-rGO NS and 284.5 eV for HfS_2_-rGO NS. Conversely, the C–C peak (sp^3^ bond) intensity is higher in HfS_2_-rGO NS due to the presence of more structural defects on C. The C–O–C peak is visible at 285.3 eV in HfP-rGO NS and at 285.9 eV in HfS_2_-rGO NS. Small peaks with binding energies centered at 286.5 eV and 287.3 eV in HfP-rGO NS may be attributed to C–OH and C=O bonds, but these were found at 287.3 eV (C–OH) and 287.8 eV (C=O) in HfS_2_-rGO NS. The O–C=O peak is seen at 288.5 eV in both HfP-rGO NS and HfS_2_-rGO NS.^42^ The increased intensity of the O–C=O peak in HfS_2_-rGO NS is likely caused by the high oxides stacked in the rGO nanosheets. The O 1s peak at 530.8 eV is obtained in HfP-rGO NS, next to that, O=C at 532.3 eV is visible easily due to the strong C and O bonding in the rGO sheets. For HfS_2_-rGO NS, O 1s is obtained at 531.6 eV and a small O=C peak^43^ is raised at 533.4 eV within the O 1s peak, which reveals that the bonding between the C and O in the rGO nanosheet is hindered.^44^

**Fig. 3:**
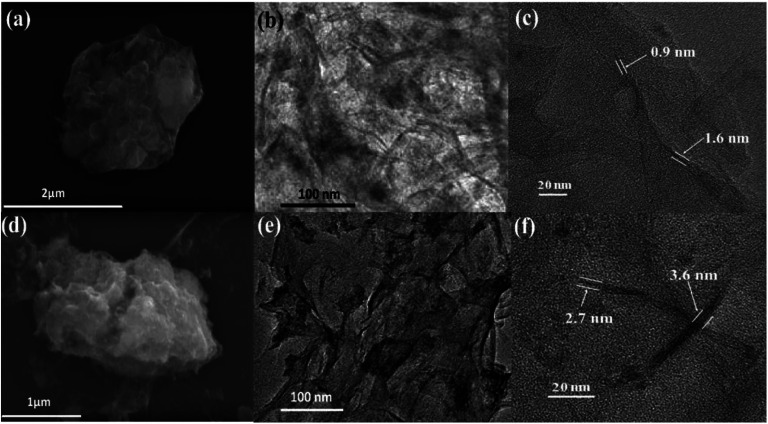
(a) SEM, (b) TEM and (c) HRTEM image of HfP-rGO nanosheets. (d) SEM, (e) TEM and (f) HRTEM image of HfS_2_-rGO nanosheets.

The SEM image of HfP-rGO NS in Fig. 3a shows the sheet-like structures, and the corresponding elemental mappings of Hf, P, C and O are shown in Fig. S2a–d,† respectively. Fig. S2† demonstrates that all elements (Hf, P, C and O) are homogeneously distributed over the sheet-like structures. Similarly, the SEM image of HfS_2_-rGO NS is shown in Fig. 3d, which reveals a sheet-like surface. The corresponding elemental mappings of Hf, S, C and O are shown in Fig. S3a–d† respectively. Similar to HfP-rGO NS, all elements (Hf, S, C and O) are distributed equally on the nanosheets, although the occurrence of O is illuminated more strongly on the elemental mapping images. Fig. 3b and e are the TEM images of HfP-rGO NS and HfS_2_-rGO NS. It can be clearly seen that the sheet-like structures are captured and no particles are detected. The high-resolution TEM images of HfP-rGO NS and HfS_2_-rGO NS are shown in Fig. 3c and f, respectively, in which thin sheets collide at their edges. The thickness of the edges in HfP-rGO NS are found to be 0.9 nm and 1.6 nm, respectively, whereas the edge thicknesses of the HfS_2_-rGO nanosheets are 2.7 nm and 3.6 nm.

### Electrochemical analysis

**Fig. 4:**
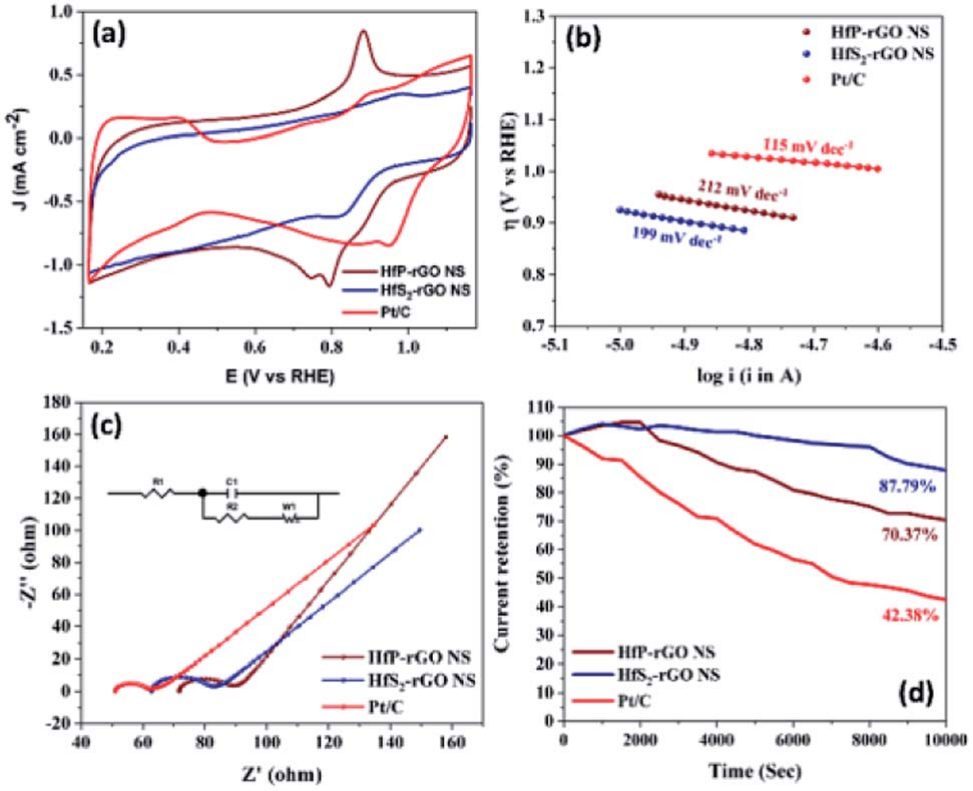
ORR electrochemical measurements of HfP-rGO and HfS_2_-rGO nanosheets: (a) cyclic voltammograms in O_2_ saturated 0.1 M KOH solution at a sweeping rate of 20 mV s^−1^, (b) Tafel plots, (c) Nyquist plots with corresponding equivalent circuits (inset) and (d) amperometric *i*–*t* curve studies.

The oxygen reduction behavior of the prepared HfP-rGO NS, HfS_2_-rGO NS and commercial Pt/C catalysts are shown as CVs in Fig. 4a. The pre-determined N_2_-saturated CVs of HfP-rGO NS, HfS_2_-rGO NS and Pt/C catalysts are shown in Fig. S4.† HfP-rGO NS catalyst started its oxygen reduction at 0.97 V *vs.* RHE (*E*_on_), whereas the onset potential (*E*_on_) of HfS_2_-rGO NS was obtained at 0.95 V. As usual, Pt/C (20 wt%) (1.05 V) is dominating on the *E*_on_ of ORR. Highly reactive P towards O ensured that the HfP-rGO NS delivered the maximum current density of 1.163 mA cm^−2^ at 0.79 V (*E*_peak_), which is higher than that of Pt/C (0.850 mA cm^−2^) and HfS_2_-rGO NS (0.612 mA cm^−2^). The peak potential (*E*_peak_) of HfS_2_-rGO NS is 0.82 V, which is higher than that of the prepared phosphide-based (HfP-rGO NS) catalyst. In general, the metal–phosphide electrocatalysts exhibit oxidation peaks during the ORR CV measurements in the alkaline testing environment, which confirms an oxidation peak by HfP-rGO NS catalyst at 0.89 V *vs.* RHE.^25^ Although Pt/C attained the highest ORR potentials (*i.e.*, *E*_on_ potential at 1.05 V and *E*_peak_ at 0.95 V), its active ORR current density (0.850 mA cm^−2^) is 27% lower than that of HfP-rGO nanosheets. Linear sweep voltammograms are shown in Fig. S5,† in that HfP-rGO NS delivered a greater ORR current than that of Pt/C and HfS_2_-rGO NS. ORR Tafel plots are shown in Fig. 4b, in which both HfP-rGO NS and HfS_2_-rGO NS had a lower overpotential (η) than that of Pt/C. Due to the impact of ORR, Pt/C achieved a lower Tafel slope value (115 mV dec^−1^)^45^ than HfP-rGO NS (212 mV dec^−1^) and HfS_2_-rGO NS (199 mV dec^−1^). The current exchange density and the electron transfer coefficient can be determined using Tafel's eqn (1):^8^1
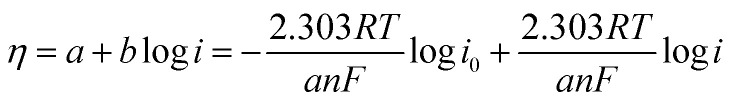
where *R* is the universal gas constant, *T* is the absolute temperature in K, *F* is Faraday's constant, *b* is the Tafel slope, *a* is the charge transfer coefficient, *n* is the electron transfer coefficient and *i*_0_ is the exchange current density. The electron transfer coefficients of HfP-rGO NS and HfS_2_-rGO NS were found to be 0.56 and 0.59, respectively. Incredibly, the exchange current density of HfS_2_-rGO NS (142.85 × 10^−3^ A cm^−2^) and HfP-rGO NS (16.24 × 10^−3^ A cm^−2^) are higher than that of Pt/C (2.8 × 10^−7^ A cm^−2^) catalyst. The catalytic activities of HfP-rGO NS, HfS_2_-rGO NS and Pt/C were further investigated by Nyquist plots, as shown in Fig. 4c. The inserted circuit model is the corresponding equivalent circuit. The ohmic resistance *R*_1_ of HfP-rGO NS, HfS_2_-rGO NS and Pt/C are 71.76, 62.76 and 51 Ω, respectively. The capacitive layer (*C*_1_) between the catalyst surface and electrolyte is found to be very thin in HfP-rGO NS (0.00095 F) compared with that of HfS_2_-rGO NS (0.00038 F) and Pt/C (0.00005 F); *i.e.*, phosphides have a thin active layer, which is two-fold bigger in the sulfides. *R*_2_, the reaction resistance between the catalyst surface and electrolyte, in multi-layered oxide-enriched HfS_2_-rGO NS shows the highest resistance of 16 Ω (*R*_2_). In contrast, Pt/C nanoparticles achieved the lowest *R*_2_ (9 Ω), whereas the median HfP-rGO obtained was 14 Ω, confirming the surface conductivity of phosphides is lower than that of sulfides. The ORR is concerned with the Warburg resistance (*W*_1_), in which both the HfP-rGO NS and HfS_2_-rGO NS catalysts achieved 16 Ω due to wide-spread catalytic centers, whereas the active Pt/C achieved 17 Ω for its hidden active spots. Fig. S6† shows the Bode plots, in which HfP-rGO displays the lowest frequency due to high electron life availability. The ORR catalytic stabilities of HfP-rGO NS, HfS_2_-rGO NS and Pt/C catalysts are shown in Fig. 4d for 10 000 s. Due to rapid degradation of Pt, the Pt/C is the main loser among the prepared catalysts, which reached 42.38% of its current at the end of the stability test. The sheet-structured HfP-rGO NS and HfS_2_-rGO NS strengthened the heteroatomic catalytic sites and maintained 70.37% and 87.79%, respectively. The early rise of the ORR current percentage is attributed to the initial reduction reaction of the available catalytic sites on the sheet towards the oxygen. The improved stability of HfS_2_-rGO was mainly due to the oxide-enriched nanosheets, whereas phosphide active centers reacted quickly towards O, causing further current loss to the HfP-rGO NS catalyst.

**Fig. 5:**
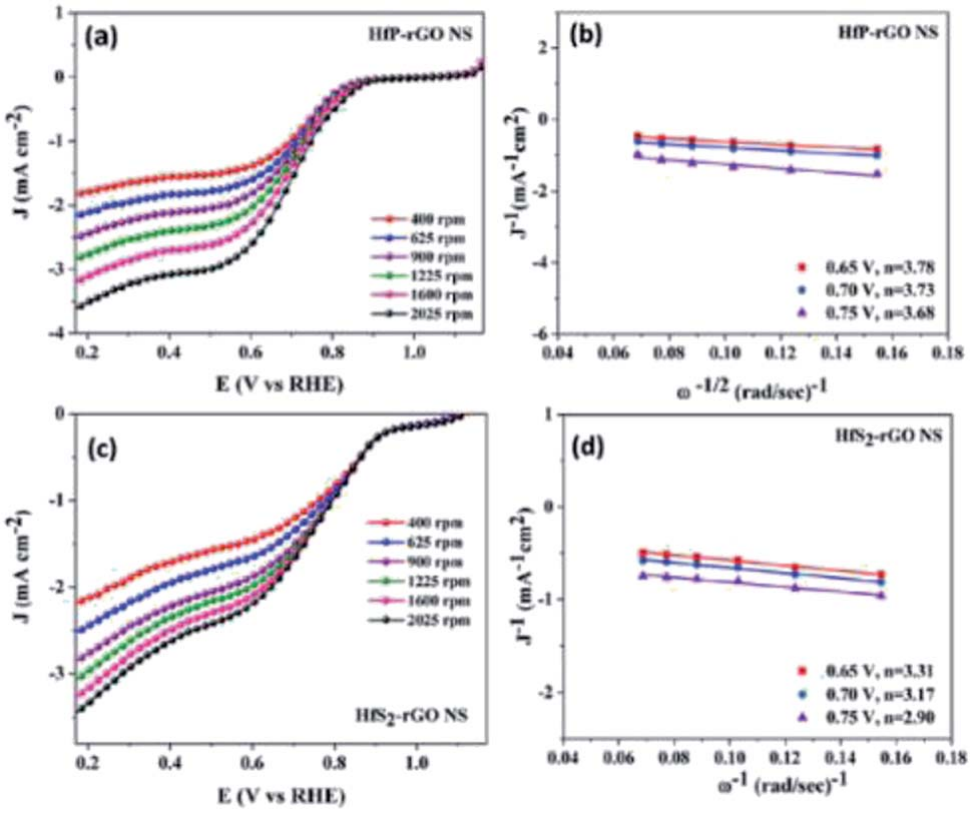
(a) Rotating disk electrode voltammograms and (b) K–L plots of HfP-rGO NS. (c) Rotating disk electrode voltammograms and (d) K–L plots of HfS_2_-rGO NS.

**Fig. 6:**
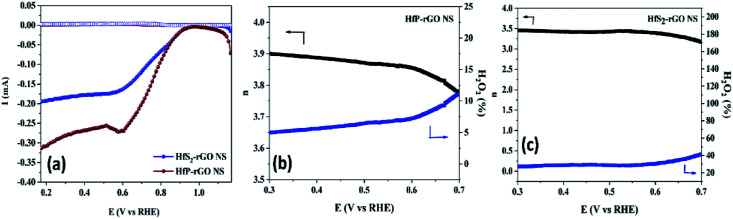
(a) Rotating ring-disk electrode voltammograms of HfP-rGO NS and HfS_2_-rGO NS. Electron transfer number ‘*n*’ and H_2_O_2_% production for (b) HfP-rGO NS and (c) HfS_2_-rGO NS.

**Fig. 7:**
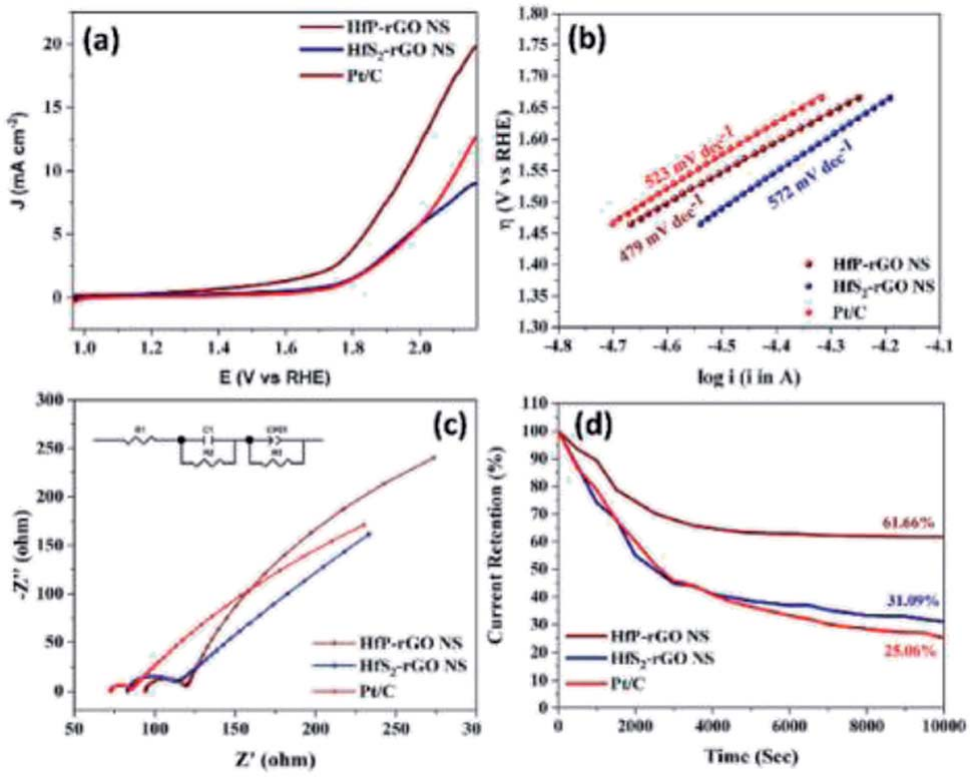
(a) OER polarization curves in 0.1 M KOH solution at a sweeping rate of 5 mV s^−1^ (rotation: 1600 rpm), (b) Tafel plots, (c) Nyquist plots with corresponding equivalent circuit (inset) and (d) amperometric *i*–*t* test results of HfP-rGO and HfS_2_-rGO nanosheets.

The electrocatalytic activity and electron kinetics of the ORR for the prepared HfP-rGO NS and HfS_2_-rGO NS catalysts were further investigated using an RDE. The RDE voltammetry curves of HfP-rGO NS and HfS_2_-rGO NS at various rotation rates are shown in Fig. 5a and c, respectively. Similar to CV, RDE onset potentials of the HfP-rGO nanosheet are higher than that of HfS_2_-rGO NS. Surprisingly, the diffusion current is well controlled by phosphides in HfP-rGO NS to fully reduce the O_2_ and to yield minimum hydrogen peroxide. Fig. 5b and d are the corresponding Koutechy–Levich (K–L) plots of HfP-rGO NS and HfS_2_-rGO NS. The following Koutechy–Levich eqn (2) is used to determine the kinetics of electrons during the ORR:2
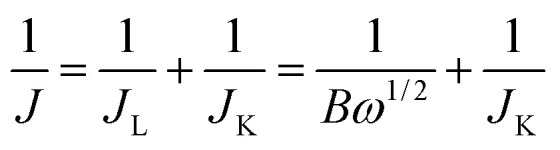
where *J* is the measured current density, *J*_K_ is the kinetic current density and *J*_L_ is the diffusion limiting current density. The angular frequency *ω* can be found using *ω* = 2*πN*/60, where *N* is the rotation rate of the working electrode.^8^ From the Koutechy–Levich equation, *B*, the slope of the K–L line can be written as following eqn (3):

3 *B* = 0.62*nFC*_0_(*D*_0_)^2/3^*ϑ*^−1/6^

where *n* is the electron transfer number, *F* is the Faraday constant, *D*_0_ is the diffusion coefficient of O_2_ in the electrolyte, *ϑ* is the kinematic viscosity and *C*_0_ is the bulk concentration of the O_2_ in the solution.^8^ Using the above equation, the electron transfer numbers (*n*) of HfP-rGO NS and HfS_2_-rGO NS are calculated at the active potential region. The *n* values in the K–L plots indicate that both HfP-rGO NS and HfS_2_-rGO NS follow the direct 4e^−^ reduction process. RRDE voltammograms of HfP-rGO NS and HfS_2_-rGO NS are shown in Fig. 6a. RRDE results once again confirm that phosphides of HfP-rGO NS are capable of delivering a higher current than the sulfides of the HfS_2_-rGO NS during the ORR. The electron transfer number and the percentage of yielded hydrogen peroxide during the RRDE tests for HfP-rGO NS and HfS_2_-rGO NS are shown in Fig. 6b and c, respectively. The electron transfer number *n* and H_2_O_2_% can be calculated using the below eqn (4) and (5):4
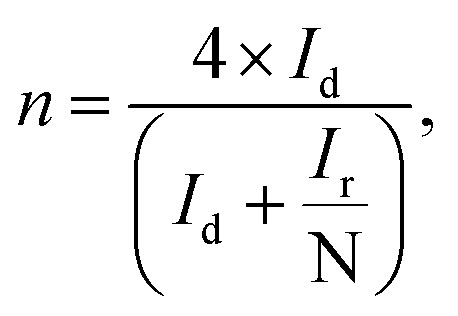
5
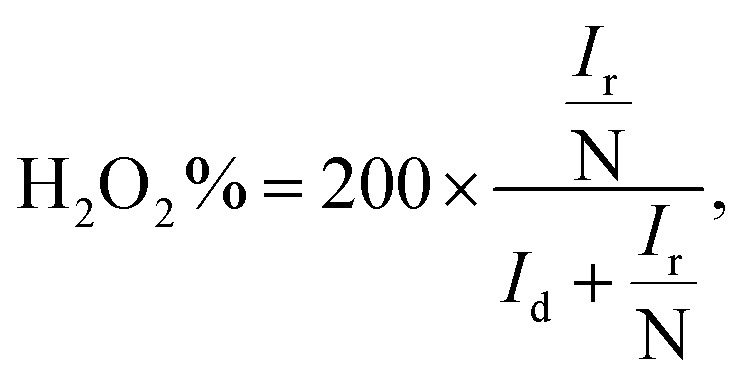
where *I*_d_ is the disk current, *I*_r_ is the ring current and *N* is the geometrical current collection coefficient (for Pt, *N* = 0.39). In the active ORR region, HfP-rGO NS had ‘*n*’ ranges between 3.75 and 3.9, but HfS_2_-rGO NS transferred electrons between 3.25 and 3.5. The H_2_O_2_% data reveal that HfP-rGO NS yielded just 5–10% of hydrogen peroxide. However, HfS_2_-rGO NS produced 30–40% of hydrogen peroxide production, which is nearly four-times higher than that of HfP-rGO NS. The oxygen evolution catalytic activities of the prepared HfP-rGO NS, HfS_2_-rGO NS and commercial Pt/C catalysts are illustrated in the polarization test, and the results are shown in Fig. 7a. HfP-rGO NS showed the highest OER current, which reached up to 19 mA cm^−2^ by the positive ion-accepting nature of phosphide to form oxygen. Both HfS_2_-rGO NS and Pt/C began oxygen evolution at 1.78 V *vs.* RHE, but HfP-rGO NS started the OER at 1.72 V with a higher current density.

To gain further insights into the highly active phosphide-based OER catalysts, HfP-rGO NS have attained the lowest potential at 1.96 V, which is lower than that of Pt/C (2.12 V) and HfS_2_-rGO NS (beyond 2.12 V) for the constant current density measurement of 10 mA cm^−2^. The OER Tafel plots of HfP-rGO NS, HfS_2_-rGO NS and Pt/C are shown in Fig. 7b. In accordance with the lower OER potentials, HfP-rGO NS achieved the lowest Tafel slope value (479 mV dec^−1^) compared with HfS_2_-rGO (572 mV dec^−1^) and Pt/C (523 mV dec^−1^). The Nyquist plots of HfP-rGO NS, HfS_2_-rGO NS and Pt/C are shown in Fig. 7c. The modulated equivalent circuit is inserted. *R*_1_ is the ohmic resistance and *R*_2_ is the reaction resistance between the electrolyte and the catalyst surface. The formed capacitive layer (*C*_1_) between the catalyst surface and the electrolyte signify a small semi-circle in the Nyquist plots. Due to the greater formation of O_2_, the capacitance (*C*_1_) of HfS_2_-rGO NS (4538 μF) is higher than that of Pt/C (545 μF) and HfP-rGO NS (357 μF). Next to the semi-circle, all catalysts have a linear curve rise that actually represents the OER resistance (*R*_3_). The efficient OER of HfP-inducted rGO catalyst is affirmed again by the least *R*_3_ value of 850 Ω. The HfS_2_-influenced catalyst (HfS_2_-rGO NS) offers three-fold higher resistance (2900 Ω). Incredibly, HfP-rGO NS again dominates over the commercial Pt/C catalyst (1028 Ω). The produced oxygen molecules on the catalyst surface imply the presence of a capacitive layer (CPE), in which HfP-rGO NS (0.0460) has a higher OER active layer than HfS_2_-rGO NS (0.1096) and Pt/C (0.1181). The OER Bode plots are shown in Fig. S7,† in which Pt/C has a maximum frequency, which means a shorter electron life,^34^ whereas HfP-rGO NS and HfS_2_-rGO NS have a better electron life due to the stable sheet-like catalyst structure. The OER catalytic stabilities of HfP-rGO NS, HfS_2_-rGO NS and Pt/C catalysts for 10 000 s are shown in Fig. 7d. Similar to the OER polarization curves, the performances of HfS_2_-rGO and Pt/C are quite similar. The nanosheet-structured HfS_2_-rGO showed better stability than Pt/C and persisted up to 31.09% of its OER current; whereas Pt/C has drained to 25.06% at the end. The active site-enriched thin HfP-rGO nanosheets were maintained at 61.66% and emerged as a stable OER catalyst.

**Fig. 8:**
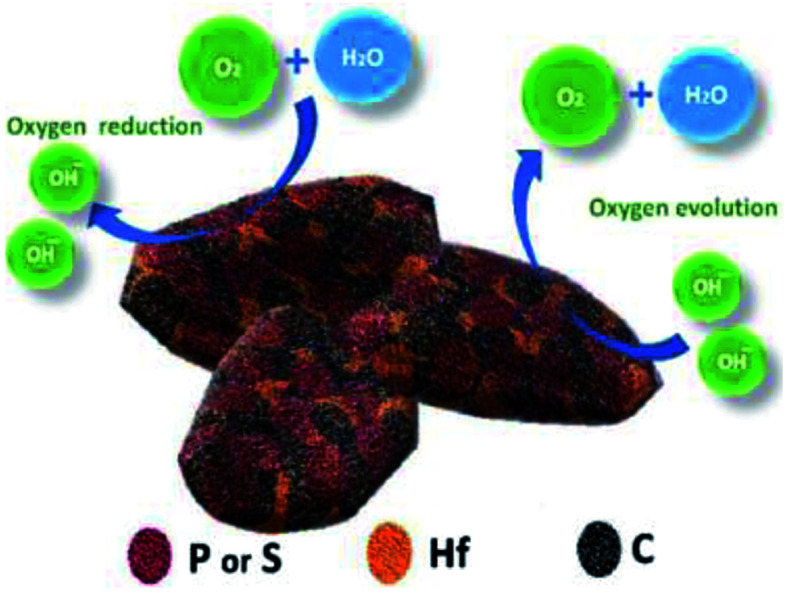
Structure and elemental arrangements of HfP-rGO/HfS_2_-rGO nanosheets, and oxygen reduction and oxygen evolution reactions.

Fig. 8 shows the structure of the prepared HfP-rGO and HfS_2_-rGO nanosheets and the bifunctional oxygen reactions. Both the HfP-rGO NS and HfS_2_-rGO NS showed superior catalytic activities compared with most catalysts for ORR and OER. For OER, at the current density of 5 mA cm^−2^, HfP-rGO NS achieved the potential at 1.82 V *vs.* RHE, which is lower than that of other catalysts such as IrO_2_ (1.85 V),^9^ RuO_2_ (1.85 V),^10^ Pt/C (1.92 V) and HfS_2_-rGO NS (1.92 V). As discussed earlier, the proton-acceptor property of phosphide-based HfP-rGO NS has an outstanding catalytic activity for O_2_ evolution. For ORR, the onset potential of HfP-rGO NS (0.97 V *vs.* RHE) is lower than that of Pt/C (1.05 V *vs.* RHE), but higher than that of HfS_2_-rGO NS (0.95 V *vs.* RHE), N/Fe-co-doped G (0.87 V),^46^ FeP@NPC (0.9 V),^31^ FeNiS_2_ NS (0.79 V),^36^ Ni_9_S_8_ NR (0.68 V),^36^ FeS NS (0.32 V),^36^ FeP@PNC-800 (0.76 V),^32^ Fe_*x*_P/NPCS (0.9 V),^25^ rGO–Co–Pi (0.91 V),^33^ Co_4_N/rGO (0.87 V)^8^ and N-CG-CoO (0.9 V)^1^ catalysts. Similarly, the ORR peak current density (*J*_peak_) of HfP-rGO NS (1.16 mA cm^−2^) is superior to Pt/C (0.85 mA cm^−2^), HfS_2_-rGO NS (0.61 mA cm^−2^), FeP@PNC-800 (0.8 mA cm^−2^),^32^ rGO–Co–Pi (0.35 mA cm^−2^)^33^ and V(C,N) (0.13 mA cm^−2^)^34^ catalysts. Furthermore, the peak potential of ORR of HfP-rGO NS (0.79 V *vs.* RHE) is higher than that of FeP@PNC-800 (0.58 V),^32^ Co_9_S_8_/G (0.69 V)^35^ and N-Co_9_S_8_/G (0.7 V).^1^ Thus, the measured electrochemical data and the discussions confirm that phosphide-based HfP-rGO nanosheets are predominant towards O_2_ evolution. For ORR, the hafnium phosphides delivered higher reaction currents, but sulfide-enriched HfS_2_-rGO NS maintained better ORR catalytic stability and higher ORR potentials at the peak to provide more energy for O_2_ reduction. The different electrocatalytic activities in HfP-rGO NS and HfS_2_-rGO NS are due to the different anions. Thus, the prepared nanosheet-like HfP and hafnium disulfide catalysts tuned by rGO sheets achieved the oxygen reactions efficiently by a synergistic effect.

## Conclusions

The influence of phosphides and sulfides in the prepared HfP-rGO NS and HfS_2_-rGO NS catalysts for ORR and OER were investigated. Due to the proton-acceptor ability, phosphide-enriched HfP-rGO NS has played a phenomenal role towards O_2_ evolution. The OER potential of HfP-rGO NS is lower than that of HfS_2_-rGO NS, Pt/C, RuO_2_ and IrO_2_, which indicates the superior catalytic activity and potential as an alternative to noble OER catalysts. During the ORR, HfS_2_-rGO NS exhibited higher catalytic stability and better Tafel slope activity; however, hafnium phosphides achieved higher ORR current density than that of HfS_2_-rGO NS and Pt/C. Also, the ORR onset potential of HfP-rGO NS is just 80 mV lower than that of the Pt/C. In particular, both the HfP-rGO NS and HfS_2_-rGO NS catalysts have achieved remarkable 16 Ω of Warburg diffusion resistance during the ORR. This is the first report concerning Hf-based bifunctional oxygen catalysts. The preparation technique and electrochemical characteristics of these prepared HfP-rGO NS and HfS_2_-rGO NS catalysts might accelerate understanding with respect to the design and development of robust oxygen catalysts.

## Conflicts of interest

There are no conflicts to declare.

## Supplementary Material

RA-009-C8RA09598A-s001
