# Supplementary material for: Electrochemical impacts of sheet-like hafnium phosphide and hafnium disulfide catalysts bonded with reduced graphene oxide sheets for bifunctional oxygen reactions in alkaline electrolytes
Source: RSC Adv. 2019 Jan 18;9(5):2599–607. doi: 10.1039/c8ra09598a (PMC9059885; doi:10.1039/c8ra09598a)
Supplement: RA-009-C8RA09598A-s001 [file RA-009-C8RA09598A-s001.pdf]

Electronic Supplementary Material (ESI) for RSC Advances.
This journal is © The Royal Society of Chemistry 2019

Electronic Supporting Information

Electrochemical impacts of sheet-like hafnium phosphide and hafnium disulphide catalysts bonded with rGO sheets for bifunctional oxygen reactions in the alkaline electrolytes

Mayilvel Dinesh Meganathan, [a] Taizhong Huang, [*a] Hengyi Fang, [a] Jianfeng Mao, [*b] and Guoxin Sun [a]

[a]School of Chemistry and Chemical Engineering, University of Jinan, 336, West Road of Nanxinzhuang, Jinan, Shandong, 250022, China.

[b]Institute for Superconducting & Electronic Materials, University of Wollongong, Wollongong, NSW 2522, Australia.

Corresponding Authors:

*Taizhong Huang, Tel +86 531-89736103, Email: chm_huangtz@ujn.edu.cn;

**Jianfeng Mao, Email: jmao@uow.edu.au

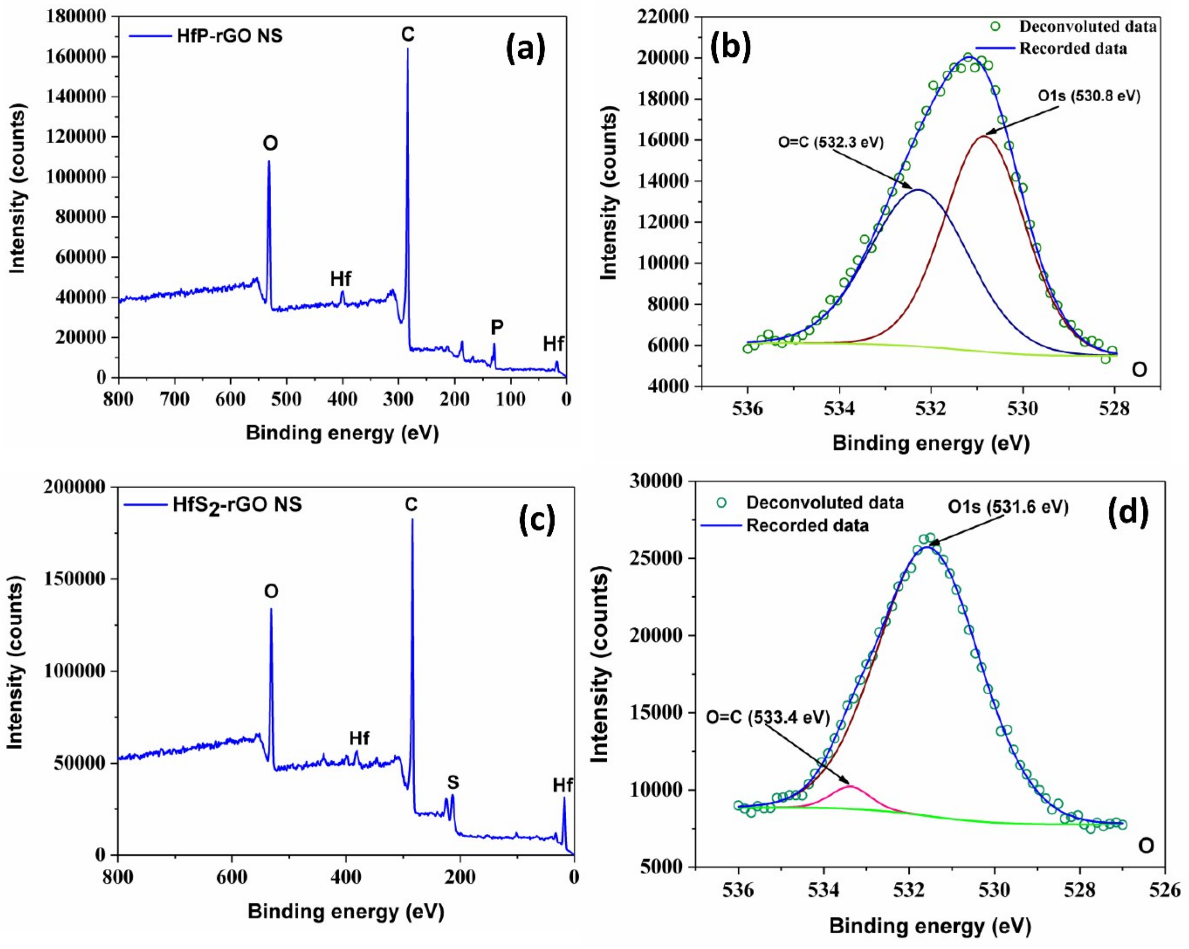

Fig. S1. (a) XPS spectra and (b) high-resolution oxygen spectra of HfP-rGO nanosheets. (c) XPS spectra and (d) high-resolution oxygen spectra of HfS_2-rGO nanosheets.

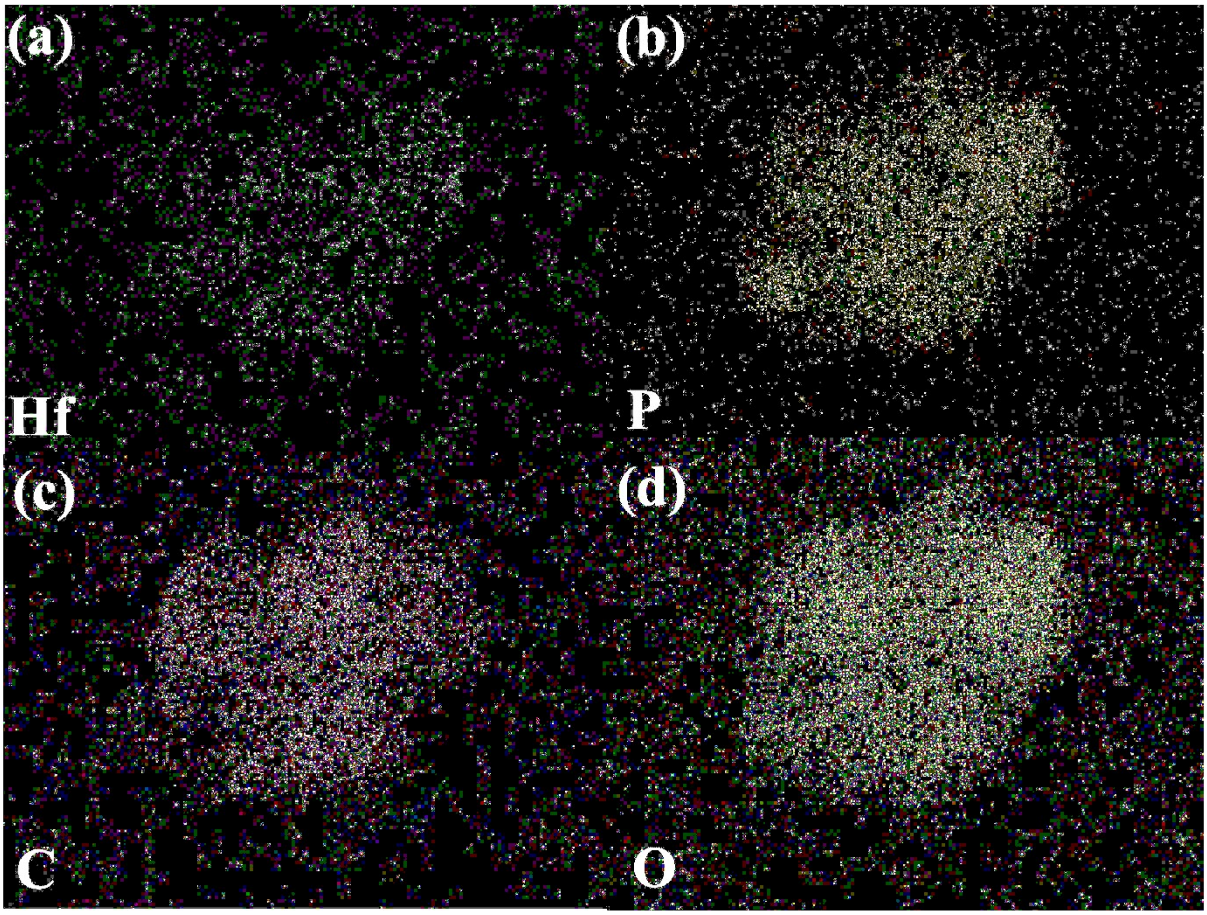

Fig. S2. SEM elemental mappings of (a) hafnium, (b) phosphorus, (c) carbon and (d) oxygen for HfP-rGO nanosheets.

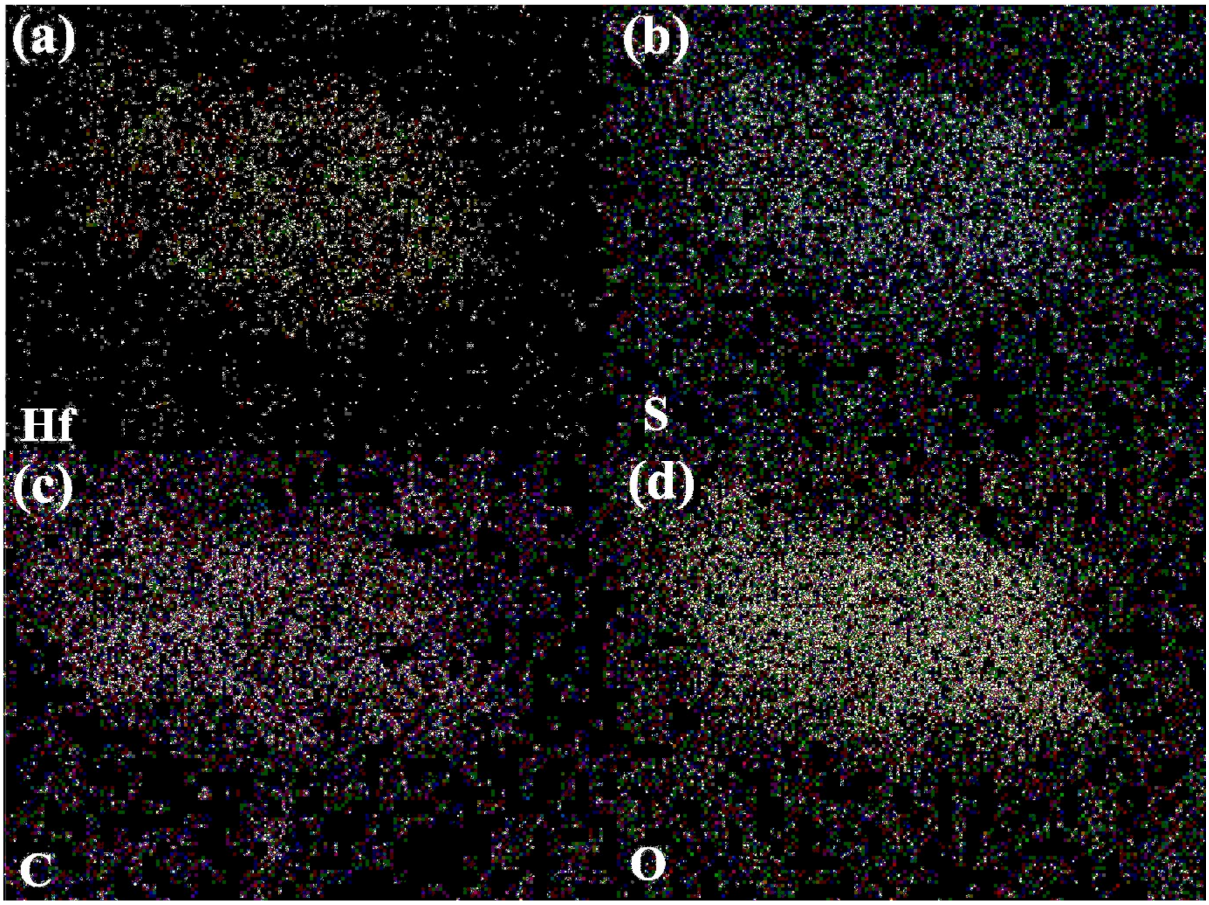

Fig. S3. SEM elemental mappings of (a) hafnium, (b) sulphur, (c) carbon and (d) oxygen for HfS_2-rGO nanosheets.

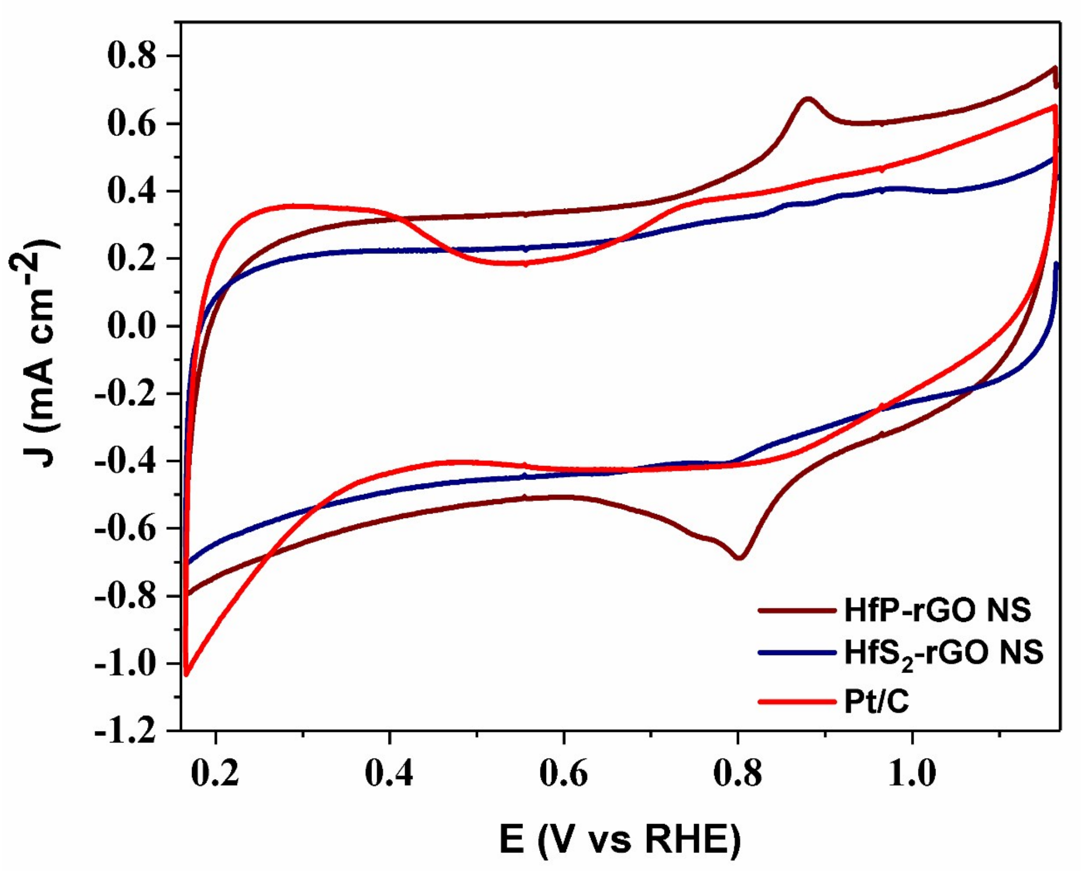

Fig. S4. Cyclic voltammograms of ORR of HfP-rGO NS, HfS_2-rGO NS and Pt/C in N_2-saturated 0.1 M KOH electrolyte at a sweep rate of 20 mV s^{-1}.

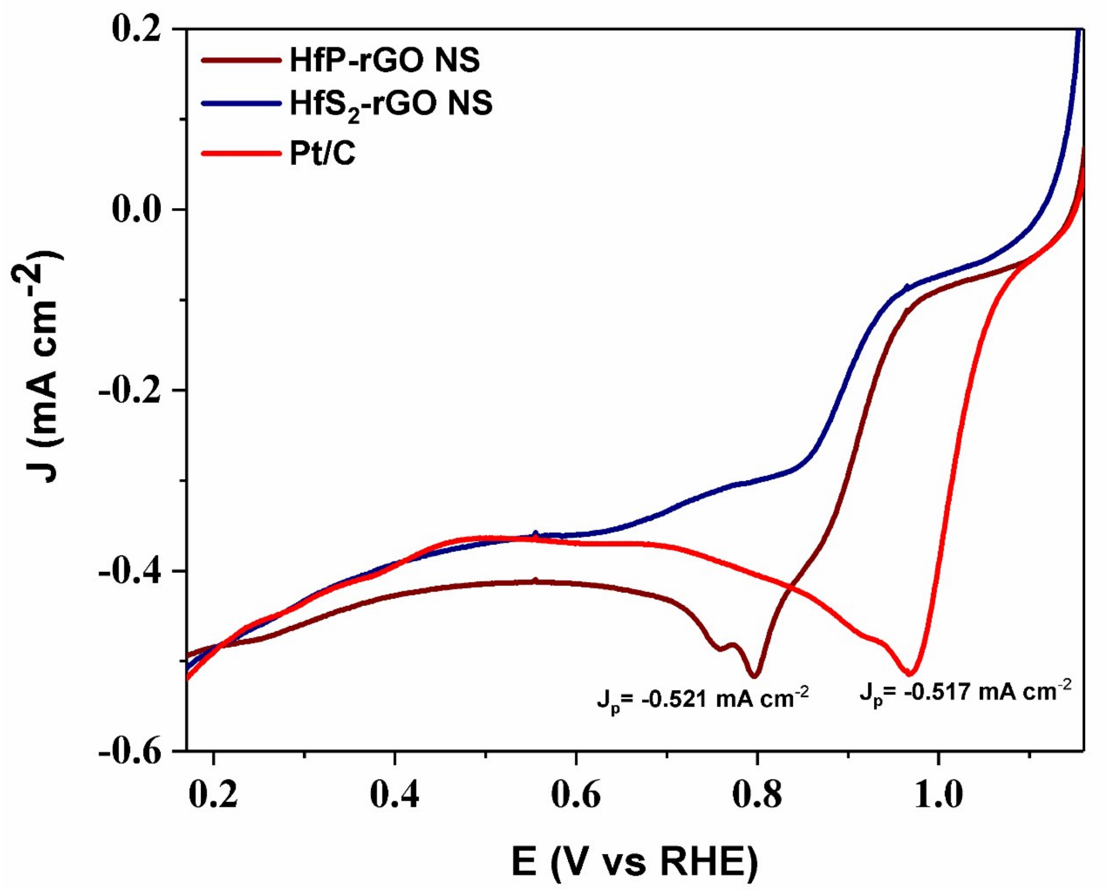

Fig. S5. Linear sweep voltammograms of HfP-rGO NS, HfS_2-rGO NS and Pt/C at a sweep rate of 5 mV s^{-1} during the ORR.

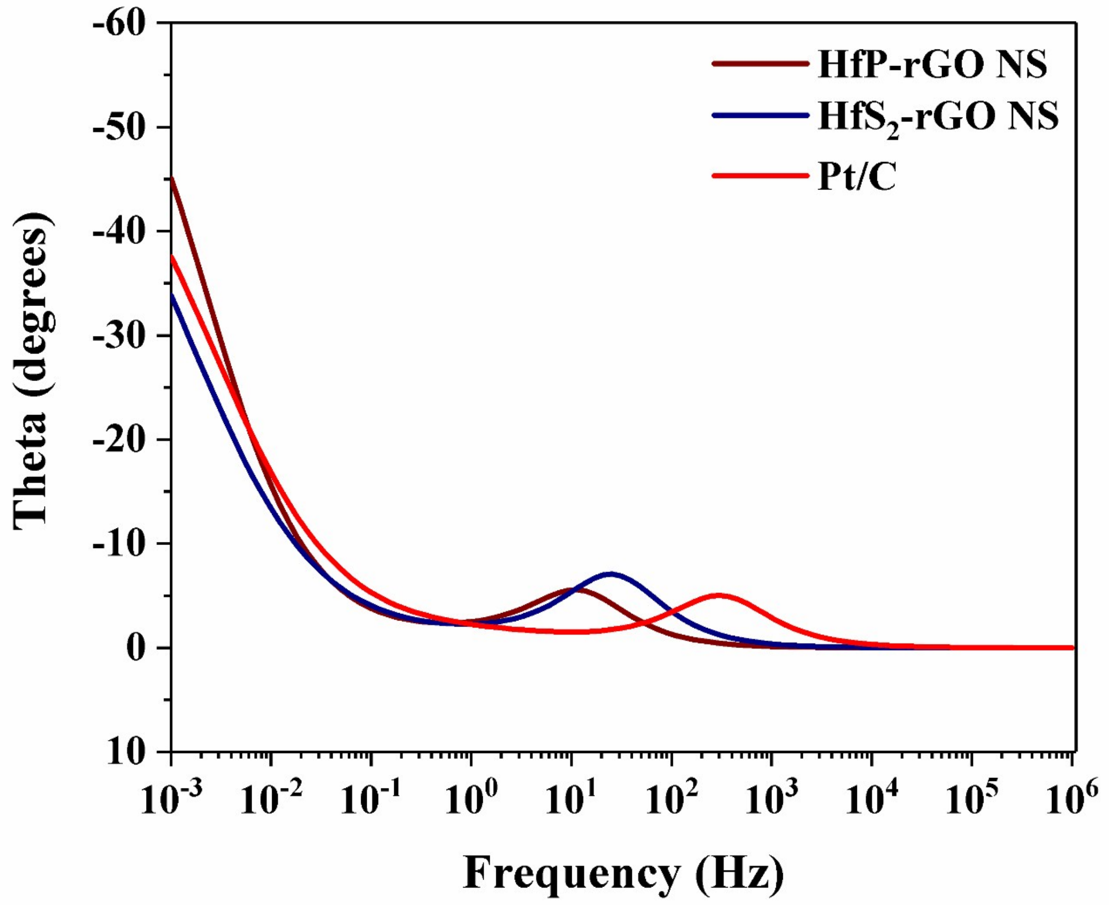

Fig. S6. Bode plots of HfP-rGO NS, HfS_2-rGO NS and Pt/C for oxygen reduction reaction.

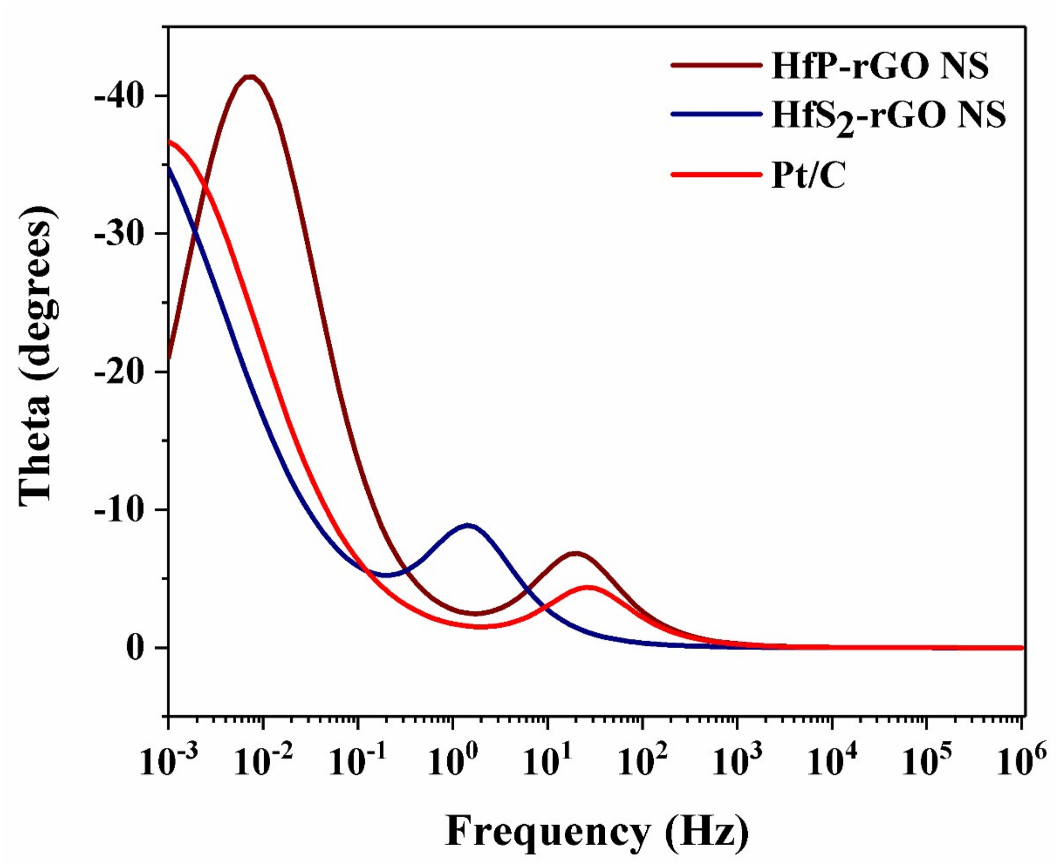

Fig. S7. Bode plots of HfP-rGO NS, HfS_2-rGO NS and Pt/C for oxygen evolution reaction.
